# Advances in vaccines: revolutionizing disease prevention

**DOI:** 10.1038/s41598-023-38798-z

**Published:** 2023-07-20

**Authors:** Timir Tripathi

**Affiliations:** Molecular and Structural Biophysics Laboratory, Department of Biochemistry, North-Eastern Hill University, Shillong, 793022 India

**Keywords:** Biochemistry, Drug discovery

## Abstract

Vaccines have revolutionized modern medicine by preventing infectious diseases and safeguarding public health. This Collection showcases cutting-edge research on advancements in vaccine development and their impact on disease prevention. The papers presented here report various facets of vaccine efficacy, immunological responses, and design, providing insight into future immunization strategies. I believe this Collection will serve as a catalyst for further advancements in the field of vaccine research.

Vaccines have long been credited as the most effective tool in preventing and managing infectious diseases. They have drastically reduced the global disease burden^1^. Over the years, significant progress has been made in understanding the immune system and developing novel vaccine design and delivery platforms^2,3^. From developing mRNA vaccines^4^ that offer rapid response to identifying novel antigenic targets for broader protection, we have been at the forefront of innovation. Furthermore, the exploration of advanced adjuvants and delivery systems is enhancing vaccine efficacy and accessibility^5^. These cutting-edge technologies and advancements in vaccine research hold immense potential for tackling infectious diseases and improving global public health. In this Collection, I am delighted to present research articles highlighting the latest advances in vaccine development, shedding light on innovative vaccine design and delivery strategies, novel targets, and promising candidates. These breakthrough articles have the potential to revolutionize the field of vaccines and move us one step closer to a world free from the grip of devastating infectious diseases and outbreaks^6^.

Early strategies for investigating new vaccine targets or developing formulations increasingly rely on sophisticated computational approaches. These approaches help save resources and refine in vitro and in vivo studies. For example, in one of the papers in this Collection, Goodswen et al.^7^ present a state-of-the-art methodology for high-throughput in silico vaccine discovery against protozoan parasites, exemplified by discovered candidates for *Toxoplasma gondii*. Vaccine discovery against protozoan parasites is challenging due to the limited number of current appropriate vaccines compared to the number of protozoal diseases that need one. The group generated a ranked list of *T. gondii* vaccine candidates and proposed a workflow integrating parasite biology, host immune system defences, and bioinformatics programs to predict vaccine candidates. Although testing in animal models is required to validate these predictions, most of the top-ranked candidates are supported by publications reinforcing the confidence in the approach.

In another paper showcasing the benefits of an in silico approach, Palatnik‐de‐Sousa et al.^8^ report the design and development of a multiepitope multivariant vaccine based on highly conserved epitopes of multiple proteins of all SARS-CoV-2 variants. The authors propose that this could offer more long-lasting protection against different strains of SARS-CoV-2 compared with current vaccines. The vaccine was developed based on highly promiscuous and robust HLA binding CD4 + and CD8 + T cell epitopes of the S, M, N, E, ORF1ab, ORF 6 and ORF8 proteins of SARS-CoV-2 variants Alpha to Omicron. The study found that the selected epitopes were 100% conserved among all 10 studied variants, supporting the potential efficacy of the multivariant multiepitope vaccine in generating cross-protection against infections by viruses of different human SARS-CoV-2 clades. The use of immunoinformatics and in silico approaches to design the vaccines in these articles could be a cost-effective and time-efficient method for developing vaccines for other infectious diseases in the future.

The translation of scientific discoveries into practical applications ensures the successful development and evaluation of effective vaccines, such as those reported by Quach et al.^9^ and Uddin et al.^10^. Quach et al.^9^ report the development of a peptide-based smallpox vaccine by identifying and evaluating immunogenic peptides from vaccinia-derived peptides. They assessed the immunogenicity of these T-cell peptides in both transgenic mouse models and human peripheral blood mononuclear cells. The vaccine, based on four selected peptides, provided 100% protection against a lethal viral challenge and induced a long-term memory T-cell response, highlighting the potential of peptide-based vaccines for infectious diseases. Uddin et al.^10^ developed and evaluated a mucosal vaccine against the bovine respiratory pathogen *Mannheimia haemolytica* using *Bacillus subtilis* spores as an adjuvant. They found that intranasal immunization of spore-bound antigens generated the best secretory IgA-specific response against both PlpE and LktA in all bronchoalveolar lavage, saliva, and faeces samples. The spore-based vaccine may offer protection in cattle by limiting colonization and subsequent infection, and Spore-MhCP warrants further evaluation in cattle as a mucosal vaccine against *M. haemolytica*. This technology has potential commercial benefits as production of *B. subtilis* is well established and has low-cost inputs, and *B. subtilis* is recognized as a probiotic that has generally regarded as safe status, used commercially in food/feed products for human beings, poultry, cattle, swine, and fish. The use of oral administration of the vaccine would allow for large-scale administration, which is especially important as livestock management strategies, including vaccination, are cost- and ease-of-use dependent. The work highlights innovative approaches to address pressing challenges in vaccine development.

Understanding cellular responses following the administration of vaccines is crucial in assessing their efficacy and safety and in the development of improved vaccine strategies. Gmyrek et al.^11^ characterize the B cell response in mice vaccinated with a live-attenuated HSV-1 mutant, 0ΔNLS, and compare it to the parental virus, GFP105. The study found that 0ΔNLS vaccination resulted in a more robust B cell response, including an increase in CD4 + follicular helper T cells, germinal B cells, and class-switched B cells, as well as an elevated titer of HSV-1-specific antibody. The study reports that HSV-1 thymidine kinase and glycoprotein M are likely expendable components in the efficacy of a humoral response to ocular HSV-1 infection. Lunardelli et al.^12^ provide a detailed assessment of the immune responses induced after immunization with different regions of the ZIKV envelope protein. The study found that immunization with E ZIKV, EDI/II ZIKV, and EDIII ZIKV proteins induced specific IFNγ-producing cells and polyfunctional CD4 + and CD8 + T cells. The study also identified four peptides present in the envelope protein capable of inducing a cellular immune response to the H-2Kd and H-2Kb haplotypes. The results suggest that the ZIKV envelope glycoprotein is highly immunogenic and could be a potential target for developing a vaccine against ZIKV. A paper by Suryadevara et al.^13^ contributes to understanding the molecular signature of CD8 + Trm cells elicited by subunit vaccination and their potential to protect against respiratory infectious diseases. The molecular signature of subunit vaccine-elicited CD8 + Trm cells resembles those elicited by virus infection or vaccination, with distinct molecular signatures distinguishing lung interstitial CD8 + Trm cells from effector memory and splenic memory counterparts. The transcriptome signature of the elicited CD8 + Trm cells provided clues to the basis of their tissue residence and function. Insights into cellular responses, such as those provided by the studies mentioned above, can not only help us understand tissue-specific responses to diseases but also how to harness them to promote resistance or treatment.

The advancements in vaccine research are transforming the landscape of disease prevention. From mRNA vaccines to novel antigenic targets, adjuvants, and delivery systems, these breakthroughs offer new avenues for combating infectious diseases and improving global public health^2,3,5,6,14,15^. Addressing vaccine hesitancy^16,17^ and ensuring equitable access to vaccines are also top priorities^18^. Continued investment in research, collaboration, and development is essential to drive innovation and overcome challenges. The Collection highlights the innovative strategies, novel technologies, and cutting-edge research in vaccine technology, formulation, and delivery systems that have revolutionized vaccine development. With these advancements, we are inching closer to a future where the burden of preventable diseases is significantly reduced, paving the way for healthier communities and a safer world.
